# Neutrophils: potential therapeutic targets in tularemia?

**DOI:** 10.3389/fcimb.2013.00109

**Published:** 2013-12-27

**Authors:** Lee-Ann H. Allen

**Affiliations:** Inflammation Program and the Departments of Internal Medicine and Microbiology, University of Iowa and the VA Medical CenterIowa City, IA, USA

**Keywords:** neutrophils, apoptosis, inflammation, *Francisella tularensis*, innate immunity

## Abstract

The central role of neutrophils in innate immunity and host defense has long been recognized, and the ability of these cells to efficiently engulf and kill invading bacteria has been extensively studied, as has the role of neutrophil apoptosis in resolution of the inflammatory response. In the past few years additional immunoregulatory properties of neutrophils were discovered, and it is now clear that these cells play a much greater role in control of the immune response than was previously appreciated. In this regard, it is noteworthy that *Francisella tularensis* is one of relatively few pathogens that can successfully parasitize neutrophils as well as macrophages, DC and epithelial cells. Herein we will review the mechanisms used by *F. tularensis* to evade elimination by neutrophils. We will also reprise effects of this pathogen on neutrophil migration and lifespan as compared with other infectious and inflammatory disease states. In addition, we will discuss the evidence which suggests that neutrophils contribute to disease progression rather than effective defense during tularemia, and consider whether manipulation of neutrophil migration or turnover may be suitable adjunctive therapeutic strategies.

## Neutrophils in innate host defense

Polymorphonuclear leukocytes (PMNs) are the most abundant leukocyte population in human blood and are rapidly mobilized to sites of infection (Kennedy and Deleo, 2009). In this locale they phagocytose microbes and utilize a combination of NADPH oxidase-derived reactive oxygen species (ROS), cytotoxic granule components and antimicrobial peptides to generate a highly lethal intraphagosomal environment (Nauseef, 2007; Kennedy and Deleo, 2009). In contrast to other leukocytes, neutrophils are short-lived and are preprogrammed to undergo constitutive (spontaneous) apoptosis 18–24 h after release into circulation, and under normal circumstances, PMN apoptosis is further accelerated by phagocytosis and oxidant production (Watson et al., 1996; Kobayashi et al., 2003c; Kobayashi and Deleo, 2009). Ground-breaking studies of DeLeo and colleagues revealed that both constitutive and infection-induced PMN death are controlled not only at the level of intracellular signaling, but also by global changes in gene expression (Kobayashi et al., 2002, 2003a,c), discoveries which necessitated revision of the long-standing notion that mature neutrophils were nearly transcriptionally inert (Jack and Fearon, 1988; Kobayashi and Deleo, 2009). Tight spatial and temporal control of PMN apoptosis is critical for elimination of infection and resolution of the inflammatory response, and during this process phagocytic and proinflammatory capacity are down-regulated, release of toxic cell components is prevented, and tissue damage is minimized (Kobayashi et al., 2003b,c; Fox et al., 2010). If this process is perturbed PMNs can develop a proinflammatory phenotype that promotes necrosis and granuloma formation and sustains infection (Kobayashi et al., 2003b, 2004). For this reason, defects in PMN turnover are indicative of an ineffective and dysregulated inflammatory response (Nathan, 2002). In keeping with this, recent studies revealed that neutrophils have immunoregulatory properties that directly influence the function of NK cells, DCs, macrophages and lymphocytes (Mantovani et al., 2011).

## Neutrophils and tularemia pathogenesis

*Francisella tularensis* is a facultative intracellular pathogen that is distributed throughout the Northern hemisphere and two subspecies of this bacterium, *F. tularensis* subspecies *tularensis* (type A) and *F. tularensis* subspecies *holarctica* (type B) account for nearly all cases of human tularemia. Most studies of this organism have focused on macrophages as major vehicles for intracellular growth and bacterial dissemination from sites of infection to the liver and spleen (Chong and Celli, 2010). Nevertheless, *F. tularensis* is unusual in its ability to infect neutrophils and epithelial cells as well as mononuclear phagocytes, but relatively little is known about the shared and distinct contributions of these other cell types to disease (McLendon et al., 2006).

Aerosol infection of rhesus monkeys with virulent type B *F. tularensis* strains defined prominent features of pneumonic tularemia, and these studies were among the first to suggest a key role for neutrophils in tissue destruction and disease progression (Tulis et al., 1970; Schricker et al., 1972; Hall et al., 1973). These data demonstrate that large numbers of PMNs are present the lungs, and from day 2 onward alveoli and bronchioles become progressively clogged with neutrophils, bacteria and necrotic debris. Granulomas also begin to organize wherein live PMNs, bacteria and necrotic debris become enveloped by epithelial syncytia. A similar disease course has been described using rats, rabbits and mice (Dunaeva and Shlygina, 1975), and *ex vivo* analyses indicate that PMNs contain viable bacteria. Rhesus monkeys, rabbits, mice (and many humans) do not survive acute infection with type A *F. tularensis* (Eigelsbach et al., 1962; Schricker et al., 1972). These organisms replicate much faster than type B isolates, and progression to moribund status is characterized by an accumulation of neutrophils and bacteria in the lung and extensive necrotic tissue damage (Conlan et al., 2003; Lamps et al., 2004; Bosio et al., 2007). In keeping with the histopathology data, flow cytometry analysis of mouse lung cells indicates that whereas alveolar macrophages account for ~70% of *F. tularensis*-infected lung cells on the first day of infection, neutrophils are the major infected cell population by day 3, and the fraction of infected DCs and alveolar type II (ATII) cells is relatively low (Hall et al., 2008).

It has long been known that neutropenia or inherited defects in PMN function markedly increase susceptibility to infection. For this reason, neutrophil-depleting antibodies are often used to determine the role of this cell type in different diseases. Results of studies that used the first antibody developed for this purpose, RB6-8C5, suggested that PMNs are critical for host defense against *F. tularensis* (Sjostedt et al., 1994). However, subsequent studies revealed that RB6-8C5 causes depletion of both inflammatory monocytes and PMNs, necessitating a reinterpretation of prior results (Daley et al., 2008; Dunay et al., 2010). A subsequent study that used a lower dose of RB6-8C5 that is somewhat more selective for PMNs did not identify a role for these cells in control of *F. tularensis* (Kuolee et al., 2011). Using a different approach, Malik et al. discovered that inhibition of PMN migration into the lung allowed mice to survive what would otherwise be a lethal dose of type A *F. tularensis* or the live vaccine strain (LVS) (Malik et al., 2007). At the same time, studies of Elkins and colleagues suggest that tularemia severity is significantly increased under conditions that induce neutrophilia, and this correlates directly with enhanced hepatotoxicity (Bosio and Elkins, 2001; Mellilo et al., 2013). On balance, the data indicate that PMNs do not contribute to effective host defense during tularemia, and instead contribute to disease progression.

## Neutrophil chemotaxis

Mechanisms of neutrophil migration to sites of infection have been extensively studied (Craig et al., 2009; Balamayooran et al., 2010; Sadik et al., 2012; Fullerton et al., 2013). In the lung, inhaled bacteria typically interact with alveolar macrophages and ATII cells, and signaling downstream of pattern recognition receptors, including TLRs, triggers secretion of major neutrophil chemotactic agents, particularly IL-8 (CXCL8) in humans and KC in mice, as well as GRO-α, MIP-2 and MCP-1. Additional players include IL-1β, LIX/CXCL5, G-CSF, complement factor C5a and the eicosanoids LTB4 and PGE2, as well as Proline-Glycine-Proline (PGP), a peptide generated from collagen in the extracellular matrix by matrix metalloproteinases MMP-8 and MMP-9. As neutrophil function and phenotype are affected by the mechanism of recruitment, it is noteworthy that IL-8, KC, GRO-α, MIP-2, LIX, and PGP all bind CXCR1 and/or CXCR2 on PMNs. Neutrophils directly contribute to this process as well via secretion IL-8, IL-1β, LTB4, and MMP-9.

**Figure 1:**
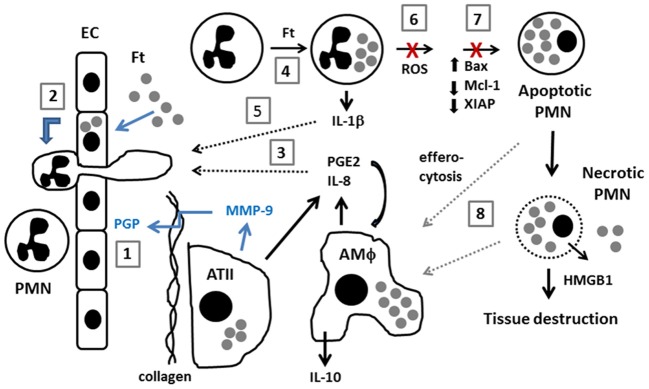
**Model of neutrophil dynamics in the *F. tularensis*-infected lung.** Inhaled *F*. tularensis rapidly infects alveolar type II (ATII) cells and macrophages (AMΦ) (Gentry et al., 2007). (1) MMP-9, likely secreted by ATII cells, cleaves collagen, generating PGP which directly stimulates PMN recruitment from the bloodstream (Malik et al., 2007). (2) PMN migration is also stimulated by direct infection of pulmonary endothelial cells (EC) by an IL-8 and MCP-1-independent mechanism (Moreland et al., 2009). (3) *F. tularensis* stimulates release of PGE2 and IL-8 from AMΦ and ATII cells (Gentry et al., 2007; Woolard et al., 2007). PGE2 stimulates macrophage production of IL-10 (Hunt et al., 2012). (4,5) *F. tularensis* infects PMNs and upregulates IL-1β (Schwartz et al., 2013). The extent to which PGE2, IL-8 and IL-1β contribute to PMN chemotaxis and phenotypic modulation during tularemia remains to be determined (*dotted black arrows*). (6,7) *F. tularensis* inhibits PMN NADPH oxidase activity and prevents changes in gene expression that are critical for constitutive and phagocytosis-induced apoptosis (McCaffrey and Allen, 2006; McCaffrey et al., 2010; Schwartz et al., 2012a, 2013). (8) Efferocytosis of apoptotic PMNs is critical for control of infection and resolution of inflammation. Defects in apoptosis favor PMN necrosis, and subsequent release of cytotoxic cell components, and danger molecules such as HMGB1 exacerbate tissue destruction. Recent data suggest that efferocytosis and/or clearance of necrotic cell debris may be impaired (Mares et al., 2011) (*dotted gray arrows*).

During tularemia a select subset of signals trigger PMN migration into the lung as the amount of IL-8 released by infected ATII cells, macrophages and endothelial cells is relatively low, and neither IL-8 nor MCP-1 appears to be essential (Gentry et al., 2007; Moreland et al., 2009). In marked contrast, *F. tularensis* specifically stimulates expression of MMP-9, and PGP is a major PMN chemoattractant in this system, as lung PMNs are markedly diminished in MMP-9 null mice and these animals are able to survive what would otherwise be a lethal dose of type A *F. tularensis* or the LVS (Malik et al., 2007), as noted above. In this regard, it is of interest that PGP is sufficient to recruit and maintain tissue neutrophils in in absence of other chemotactic agents and its stability and activity are enhanced by N-acetylation (Snelgrove et al., 2010), but whether this modification is induced during tularemia is unknown. PGE2 is produced by *F. tularensis*-infected macrophages and ATII cells (Woolard et al., 2007) and infected PMNs upregulate IL-1β (Schwartz et al., 2013), but the extent to which these agents contribute to PMN chemotaxis and phenotypic modulation during tularemia remains to be determined. A model that integrates these data is shown in Figure 1.

## *F. tularensis* disrupts neutrophil defense mechanisms

*Francisella tularensis* lipopolysaccharide (LPS) has an atypical structure that does not signal through toll like receptors 4 or 2. Nevertheless, LPS O-antigen and capsular polysaccharides act in concert to protect this organism from the lytic effects of serum complement (McLendon et al., 2006). This is significant as neutrophil uptake of *F. tularensis* is markedly enhanced by complement factors in normal human serum, and under these conditions phagocytosis is mediated by complement receptor 1 and complement receptor 3 (Schwartz et al., 2012b). Neutrophil receptors that confer inefficient phagocytosis of unopsonized *F. tularensis* remain obscure.

Under normal circumstances, phagocytosis is coupled to rapid activation of the Nox2 NADPH oxidase complex, such that toxic oxidants kill most bacteria within 60 min of infection (Deleo et al., 1999; Kobayashi et al., 2003a; Nauseef, 2007). In marked contrast, we and others demonstrated using several strains of type A and type B *F. tularensis* as well as LVS that this organism does not trigger a respiratory burst in human or monkey PMNs (Proctor et al., 1975; McCaffrey and Allen, 2006; McCaffrey et al., 2010). At the molecular level, forming *F. tularensis* phagosomes exclude flavocytochrome b558, which contains the catalytic core of the NADPH oxidase and also acts as a docking site in the membrane for the cytosolic subunits that are also essential for ROS production (McCaffrey and Allen, 2006; McCaffrey et al., 2010). Within minutes, the ability of infected PMNs to be activated by heterologous particulate and soluble stimuli is also profoundly impaired, and in this case *F. tularensis* acts at a later stage to inhibit the activity of enzyme complexes at the membrane (McCaffrey and Allen, 2006; McCaffrey et al., 2010). A similar, if not identical, mechanism of post-assembly NADPH oxidase inhibition also undermines the ability of specific IgG in anti-*F. tularensis* immune serum to enhance bacterial killing via neutrophil activation (McCaffrey and Allen, 2006; McCaffrey et al., 2010).

Evasion of toxic oxidants at early stages of infection is followed by phagosome egress, and release of bacteria into neutrophil cytosol, and *F. tularensis* replicates to some extent in this locale, though but not to the same extent as in macrophages (McCaffrey and Allen, 2006; Schulert et al., 2009; Schwartz et al., 2012a; Long et al., 2013). FevR and MigR, major virulence regulators of *F. tularensis*, are essential for inhibition of neutrophil activation (Buchan et al., 2009; McCaffrey et al., 2010). The genes in the MigR/FevR regulon required for NADPH oxidase inhibition are as yet unknown, but are distinct from pathogenicity island genes required for phagosome escape and intracellular growth such as *iglI* and *iglJ* (McCaffrey et al., 2010; Long et al., 2013). Other genes such as *carA, carB*, and *pyrB* act indirectly via effects on pyrimidine biosynthesis (Schulert et al., 2009), and the role of the acid phosphatase AcpA in NADPH oxidase inhibition and virulence is controversial (Reilly et al., 1996; Child et al., 2010; McCaffrey et al., 2010; Mohapatra et al., 2010).

## *F. tularensis* prolongs neutrophil lifespan via effects on signaling and gene expression

Neutrophils are short-lived and undergo constitutive apoptosis at a rate of 10^11^ cell per day in humans (Kennedy and Deleo, 2009). Although the specific events that initiate constitutive PMN apoptosis are unknown, it is clear that cell death is tightly regulated and that both intrinsic and extrinsic pathway caspases contribute to this process along with complex changes in gene expression that comprise and “apoptosis differentiation program,” as noted above. Since the seminal studies of Watson (Watson et al., 1996) it has also been clear that phagocytosis and NADPH oxidase-derived ROS profoundly accelerate PMN apoptosis as compared with unstimulated controls (Kobayashi et al., 2003a, 2004). This “phagocytosis-induced cell death” (PICD) respone is also regulated at the level of gene expression, and is critical for effective host defense. Blockade of NADPH oxidase activity by *F. tularensis* suggested that this organism may not accelerate PMN death, and as such may not induce PICD. Indeed, our recent biochemical studies and analysis of PMN gene expression provide definitive evidence that *F. tularensis* not only fails to induce PICD, but also inhibits constitutive neutrophil apoptosis via effects on the intrinsic and extrinsic pathways, and in this manner profoundly prolongs cell lifespan (Schwartz et al., 2012a, 2013)

Specifically, the biochemical data indicate that the vast majority of infected PMNs do not progress to an apoptotic morphology within 48 h of infection with live *F. tularensis*, and in keeping with this PS externalization, processing and activation of caspases-8, −9, and −3, and DNA fragmentation are markedly impaired (Schwartz et al., 2012a). At the same time, *F. tularensis* significantly alters the expression of over 3400 human neutrophil genes between 3 and 24 h of infection, including 365 unique genes linked to apoptosis and cell survival (Schwartz et al., 2013). Of particular note are effects of *F. tularensis* on BAX, a proapoptotic member of the Bcl-2 family of proteins that plays a pivotal role in the intrinsic apoptotic pathway via disruption of the outer mitochondrial membrane. Upregulation of BAX is a hallmark of the PICD response (Kobayashi et al., 2002, 2003a, 2004). However, *BAX* mRNA and protein are progressively downregulated by *F. tularensis* (Schwartz et al., 2013). It is therefore likely that blockade of the respiratory burst and downregulation of BAX synergize to prevent PICD during *F. tularensis* infection. At the same time, upregulation of several prosurvival factors and anti-apoptosis genes collaborate to diminish and delay the constitutive apoptosis program in PMN. Although not all of these data can be discussed here, sustained expression *BIRC4*, which encodes X-linked inhibitor of apoptosis protein (XIAP), and *CAST* which encodes calpastatin, likely account in large part for defective processing and activation of intrinsic pathway caspases (Schwartz et al., 2013). At the same time not all genes associated with enhanced PMN survival are modulated by this pathogen, as expression of IL-8 (CXCL8) is not induced and this cytokine is not secreted by PMNs infected with live *F. tularensis* (Schwartz et al., 2012a, 2013). In contrast, IL-8 appears critical for sustained survival of neutrophils infected with the obligate intracellular pathogen *Chlamydia pneumoniae* (van Zandbergen et al., 2004), and as such the data indicate that these two pathogens use distinct mechanisms to modulate PMN lifespan.

It is also important to note that apoptosis normally downregulates PMN functional capacity, and if apoptosis is inhibited these cells exhibit a sustained proinflammatory phenotype (Kobayashi et al., 2003b; Kennedy and Deleo, 2009). Consistent with this, *F. tularensis*-infected PMNs show enhanced expression of *VEGF, IL6, IL1B, CXCL1, OSM*, and *IL1RN* (Schwartz et al., 2013). Together, enhanced lifespan and proinflammatory capacity increase the probably of cell progression to secondary necrosis, which is characterized by spilling of DAMPs, alarmins, and other cytotoxic molecules that further amplify inflammation and cause extensive host tissue destruction (Kobayashi et al., 2003c; Fox et al., 2010; Silva, 2010).

## Targeting pmns as a candidate adjunctive theraputic strategy

Neutrophil accumulation and enhanced longevity, granuloma formation, and extensive tissue necrosis are benchmarks that define a defective inflammatory response (Nathan, 2002), and are also characteristic features of tissues infected with *F. tularensis* (Tulis et al., 1970; Schricker et al., 1972; Hall et al., 1973). The fact that blockade of PMN influx into the lung favors host survival without markedly altering tissue bacterial load (Malik et al., 2007) is consistent with an immunoregulatory role for PMNs in tularemia pathogenesis (Mantovani et al., 2011), as are the effects of *F. tularensis* on neutrophil activation state, lifespan and proinflammatory capacity that are noted above and summarized in Figure 1. Considered together, the data support a model in which neutrophils play a unique role in tularemia pathogenesis via dysregulation of the inflammatory response that is distinct from the role of macrophages as major vehicles for bacterial growth and dissemination. Thus, suitable points of therapeutic intervention may include PMN chemotaxis, apoptosis or activation state.

Inhibition of PMN apoptosis and aggressive neutrophilic inflammation also exacerbate the severity of pneumococcal meningitis (Koedel et al., 2009) and contribute significantly to lung destruction in human patients with chronic obstructive pulmonary disease (COPD) (Weathington et al., 2006). As in tularemia, PMN accumulation in the lungs of humans with COPD is driven by MMP-9-dependent production of PGP, and published data suggest that targeting this pathway with anti-PGP antibodies suppresses neutrophil responses and appears to have some therapeutic benefit in mouse COPD models (Weathington et al., 2006). Similarly, Arginine-Threonine-Arginine peptides block IL-8 and PGP signaling at the level of CXCR1 and CXCR2 to inhibit PMN migration and activation, and in this manner ameliorate lung destruction during emphysema (van Houwelingen et al., 2008). Other interventions that directly target this mechanism of PMN chemotaxis include small molecule inhibitors of MMPs such as GM6001, CP-471,474, and RS113,456 and enhancement of endogenous mechanisms of MMPs inhibition via intratracheal delivery of recombinant TIMPs (tissue inhibitors of metalloproteinases) (Djekic et al., 2009). As protein-based therapeutics are very expensive they are not optimal for treatment of chronic illnesses, yet could be of considerable benefit in the context of acute infectious diseases, including tularemia.

Other potential therapeutic targets include lipid mediators of the eicosanoid family, which are dysregulated in many critical illnesses. In particular, resolvin E1 is of interest as it is effective at concentrations as low as 1 nM, is beneficial for treatment of aspiration pneumonia, and is known to decrease lung PMNs via effects on ROS production, PICD and efferocytosis (Fullerton et al., 2013). Aspirin-triggered resolvins also appear to reduce mortality associated with systemic inflammatory response syndrome. On the other hand, excess or sustained production of PGE2 by macrophages and epithelial cells induces a state of “injurious resolution” that compromises Fc receptor function and NADPH oxidase activity and alters macrophage phenotype. This condition can occur in burn patients or infection with *Aspergillus*, and is reversed in by aspirin or cyclooxygenase inhibitors which reduce PGE2 levels by 95% (Fullerton et al., 2013). Although further analysis of eicosanoid profiles during tularemia is needed, these lipid mediators are of interest as PGE2 is induced by *F. tularensis* and dampens at least some aspects of the immune response (Woolard et al., 2007).

Other studies have begun to examine the potential therapeutic utility of PMN apoptosis induction. Cyclin-dependent kinases (CDKs) regulate growth of most cell types, yet are critical regulators of PMN viability and lifespan (Witko-Sarsat et al., 2011). Inhibition of CDKs with R-roscovitine and other related compounds can induce apoptosis and resolution of PMN-dominant inflammatory responses (Leitch et al., 2010), and in this manner accelerate recovery of mice with pneumococcal meningitis (Koedel et al., 2009). Moreover, our recent data indicate that CDK7 and CDK2 are induced in PMNs by *F. tularensis* (Schwartz et al., 2013). In summary, given the potentially short widow between the onset of symptoms, severe disease and death, future studies should consider whether agents that modulate PMN chemotaxis, directly target PMN apoptosis, or modulate the inflammatory response may be useful as adjunctive therapeutic agents when combined with antibiotics for treatment of tularemia.

## Relevance to other infectious diseases

As in tularemia, PMNs are the most commonly infected cell type in the airway of persons infected with *Mycobacterium tuberculosis* (Lowe et al., 2012). PMN lifespan is prolonged, and protective CD4+ T cell-driven adaptive immune responses are curtailed at the level of DCs, as downstream responses are less efficient when these cells are directly infected than when antigens are acquired by efferocytosis of apoptotic, infected PMNs (Blomgran and Ernst, 2011). Thus, neutrophilia is associated with impaired control of infection and correlates directly with the severity of cavitary disease and tissue damage. Neutrophils may kill some *M. tuberculosis* early in infection, but PMN depletion at later stages appears to be beneficial (Lowe et al., 2012). Many factors drive PMN accumulation during tuberculosis including macrophage and DC-derived IL-8, G-CSF, and LTB4, as well as IL-1β, IL-8, and LTB4 from PMNs themselves (Lowe et al., 2012). A role for PGE2 has also been described, and MMP-9 may play a specific role in recruitment of PMNs to granulomas (Lowe et al., 2012; Hawn et al., 2013).

*Brucella abortus* replicates in an ER-derived vacuole in macrophages. Similar to *F. tularensis, B. abortus* LPS has low bioactivity, yet in contrast to *F. tularensis*, there are relatively few PMNs in the circulation or at sites of infection (Barquero-Calvo et al., 2007). At the same time, *B. abortus* is not efficiently killed by human PMNs *in vitro* (Kreutzer et al., 1979), and neutrophils do not appear to contribute directly to bacterial killing. Rather, control of infection is favored by PMN depletion during late stages of infection, which enhances lymphocyte activation and proinflammatory cytokine production ((Barquero-Calvo et al., 2007, 2013). Arthritis is a complication of *B. abortus* infection, and local secretion of MMP-2 and MMP-9 plays an important role in neutrophil recruitment and joint damage (Scian et al., 2011). In contrast, C5a, IL-1β, and LTB4 play dominant roles in neutrophil-mediated joint damage in rheumatoid arthritis (Sadik et al., 2012).

Finally, MMP-8 and MMP-9 also drive PMN recruitment to the lungs during cystic fibrosis, and this is perpetuated and amplified by MMP-9 released by PMN degranulation. PGP, particularly in its highly active N-acetylated form, is elevated in patient sputum (Xu et al., 2011). Thus, strategies that target MMPs may also be useful in this disease that is characterized by chronic neutrophilic inflammation. Considered together, the data summarized here demonstrate that PMNs play complex and important roles in infection, and therapeutic strategies that target this cell type are being developed and may have wide utility alone or in combination with antibiotics.

### Conflict of interest statement

The author declares that the research was conducted in the absence of any commercial or financial relationships that could be construed as a potential conflict of interest.

## Abbreviations

ATII: alveolar type II cells
CDK: cyclin-dependent kinase
COPD: chronic obstructive pulmonary disease
DAMPs: danger-associated molecular patterns
LVS: live vaccine strain
MMP-9: matrix metalloproteinase-9
PICD: phagocytosis-induced cell death
PMN: Polymorphonuclear leukocyte
ROS: reactive oxygen species.
